# Handgrip Based Action Information Modulates Attentional Selection: An ERP Study

**DOI:** 10.3389/fnhum.2021.634359

**Published:** 2021-03-04

**Authors:** Sanjay Kumar, M. Jane Riddoch, Glyn W. Humphreys

**Affiliations:** ^1^Department of Psychology, Oxford Brookes University, Oxford, United Kingdom; ^2^Department of Experimental Psychology, University of Oxford, Oxford, United Kingdom

**Keywords:** affordance, attention, ERP, N2pc, topdown attention

## Abstract

Prior work shows that the possibility of action to an object (visual affordance) facilitates attentional deployment. We sought to investigate the neural mechanisms underlying this modulation of attention by examining ERPs to target objects that were either congruently or incongruently gripped for their use in the presence of a congruently or incongruently gripped distractor. Participants responded to the presence or absence of a target object matching a preceding action word with a distractor object presented in the opposite location. Participants were faster in responding to congruently gripped targets compared to incongruently gripped targets. There was a reduced N2pc potential when the target was congruently gripped, and the distractor was incongruently gripped compared to the conditions where targets were incongruently gripped or when the distractor, as well as target, was congruently gripped. The N2pc results indicate that target selection is easier when action information is congruent with an object’s use.

## Introduction

Our attentional system is designed to optimize functionally relevant selection for action (Alport, 1987). Following this, the brain analyses competing objects based on multiple visual properties including whether the stimuli suggest (afford) an action (Duncan et al., 1997). The effects of an affordance on a visual selection are shown dramatically in neuropsychological studies of visual extinction. Patients with extinction can identify a single object in the contralesional field but show reduced awareness for the same item when it appears simultaneously with an ipsilesional stimulus. Riddoch et al., 2003) demonstrated that extinction was reduced if the contra- and ipsilesional objects appeared to interact in a familiar action (e.g., a bottle pouring into a glass), compared to when the same objects did not interact. Subsequent work has established that this affordance effect on attentional selection is modulated by various factors including the familiarity of the action and matches between the spatial positions of the objects and the hands the patients would have used pre-morbidly for interacting with the objects (Riddoch et al., 2006; Humphreys et al., 2010, 2013).

Other neuropsychological data show that preparing action to an object can facilitate attention to an object appropriate for the prepared action. Action preparation can reduce visual neglect when the object is presented on the contralesional side of space (Humphreys and Riddoch, 2001). Similar findings can be demonstrated in normal participants. For example, Yoon et al. (2010) report that judgments about whether objects are used together are facilitated if the objects are shown being grasped using the usual hands for action seen from the observer’s point of view. Furthermore, Roberts and Humphreys (2010) have shown that positioning objects for action modulate brain activation in brain regions associated with object recognition (e.g., lateral occipital cortex) even when the objects are not attended. Bekkering and Neggers (2002) and Forti and Humphreys (2004) have further demonstrated the effects of action preparation, which biases attention to properties of objects that match the action. In the experiment by Bekkering and Neggers (2002), participants had to either look and point at a predefined target amongst distractors or look at and grasp the target. Participants made fewer saccades to target objects with wrong orientation in grasping condition than pointing condition. The effects of action planning on visual search have further been demonstrated by Feldmann-Wüstefeld and Schubö (2015) wherein the participants were instructed to plan a pointing or grasping movement which was followed by a cue to remember. Participants had to perform a visual search task while holding the cue in their memory. After the search task, a display appeared which contained the memory cue and other distractors. At this stage, participants performed the planned action (pointing or grasping) towards the cue held in memory. The results showed that attentional deployment was more pronounced [shorter Reaction times (RTs)] when participants prepared a grasping movement suggesting a closer link between action, attention, and working memory. The effect of the appropriateness of action planning on attention has been investigated by Yoon and Humphreys (2005) who presented healthy participants with single objects or non-objects which were either grasped in the normal way for action or they were assigned the grasp for another object that was inappropriate for action with the presented stimulus. The task was to decide whether the depicted stimulus was an object or a non-object. Although the grasp response was irrelevant to the task, the performance was affected by the congruency of the grasp. The performance was faster when the grasp was congruent compared with when it was incongruent with the object. This effect of grasp congruency could reflect the visual familiarity of the congruent grasp or a motor response generated about the perceived action. Kumar et al. (2013) assessed this using electroencephalography (EEG). They measured event-related desynchronization in the mu frequency above the motor scalp area as an indication of response preparation. They found greater desynchronization to congruently grasped objects with a peak in desynchronized power occurring quite early after stimulus presentation (100 ms). The result is consistent with performance being modulated by a rapid motor-based response to the congruent grip.

Electrophysiological evidence of attentional allocation from object-directed grasping action is varied. For example, Handy et al. (2003) reported that early components of the event-related potential (ERP) response (e.g., the P1) were enhanced when an action-related object fell in the lower right visual field. The authors argued that this was the location where actions would normally be addressed to the stimulus. Similarly, Freeman et al. (2016) have shown that affording objects modulate the P1 ERP component linked with early visual attention processing. Goslin et al. (2012) have also shown that when the handle of the object is oriented to afford action, there is enhanced attentional deployment indexed through modulation of the P1 and N1 ERP components. However, while examining the P1 and N1 effect of affordances on attention, Lien et al. (2013) did not find evidence of attentional modulation from intended object-directed grasping action. The authors used stimuli from Goslin et al. (2012) and presented objects centrally or peripherally (in different experiments) to the fixation point and observed that the attention-sensitive P1 and N1 components were modulated only when objects were placed peripherally suggesting spatial information rather than affordance information is significant in guiding attention.

It has already been demonstrated that the perception of graspable objects activates regions of parietal and prefrontal brain regions (Grafton et al., 1996; Chao and Martin, 2000; Grèzes and Decety, 2002) and the activation of the anterior supramarginal gyrus (SMG) area has been linked to attributes associated with grasping and manipulating tools (Johnson-Frey et al., 2005). Grasping responses to objects have been shown to activate the anterior intraparietal sulcus (aIPS; Fogassi et al., 2001; Valyear et al., 2007). Thus, activation of these motor-related areas may reflect the retrieval of stored attributes associated with grasping and manipulating tools (Chao and Martin, 2000). Furthermore, neuroimaging evidence suggests that visuomotor and attentional control systems have a common projection from precentral brain regions (see Handy et al., 2003, for a discussion), hence visuomotor processing is likely to affect the orienting of spatial attention (Handy et al., 2003) through their fMRI data showed that tools grabbed spatial attention only when tools activated premotor and prefrontal cortices, the brain regions critical for visually guided action and their planning.

In the current study, we evaluated whether a congruently grasped object also attracted attention as a function of whether targets and distractors were grasped correctly by examining ERPs. Typically, in a visual environment, multiple objects compete for our attention and our attentional system selects relevant information in a goal-directed fashion? (Johnston and Dark, 1986). The attentional selection mechanism, where irrelevant salient distractors can also capture attention in a bottom-up stimulus-driven manner (Theeuwes, 1991, 1992), incorporates a mechanism to suppress irrelevant distracting information (Desimone and Duncan, 1995). The interplay between target and distractor is a complex process where attention to the target is also affected by the features shared between the target and distractors (Duncan and Humphreys, 1989; Avraham et al., 2008).

Previous studies examining the effects of object affordance where P1 and N1 ERP components are sensitive have mainly used a single object as a target. Our interest was focused on the N2pc response as this component provides a unique online marker of the selective attentional processing of targets in presence of distractors (Kiss et al., 2007). The N2pc is a negative deflection emerging 200–300 ms post-stimulus presentation at the posterior brain position contralateral to the evoking stimulus. This component reflects the efficiency of visual selection (Kiss et al., 2008) and co-varies with the neural competition for selection (Luck et al., 1997). Notably, the N2pc is found to be higher for more attention-demanding tasks (Luck and Hillyard, 1994a). According to Luck’s theory, N2pc amplitude reflects a spatial filter mechanism to attenuate the processing of information from irrelevant surrounding distractors. Telling et al. (2010) reported a smaller N2pc component for trials when a related semantic distractor fell on the opposite side to the target compared to when the related semantic distractor fell on the same side to the target. This N2pc effect showed a shorter RT for opposite side target-distractor trials compared to same side target-distractor trials. These findings suggested that the N2pc amplitude reflects the ease of selecting a target as the N2pc is the sum of two independent selection processes, one associated with the target processing and the other with the distractor processing. This account of N2pc has also been supported by Hickey et al. (2009) in a set of experiments using the lateralized or vertical meridian presentation of target and distractors Hickey et al. (2006) isolated two components: (1) related to target processing (NT); and (2) related to distractor suppression (PD). The N2pc was the sum of these two components with both contributing to the target selection. Here, we assessed if the N2pc had a greater amplitude when the target had an incorrect grasp and the distractor a correct grasp (when target selection should be hardest), compared with when the target was grasped correctly, and the distractor grasp was incorrect. Such a result would indicate the effect of grasp congruency on visual selection. The processing of the target and distractor would be reflected in the N2pc activity, which is the sum of NT and PD. Here enhanced PD related to suppressing a more interfering distractor (distractor with a congruent grasp) will be reflected in a larger N2pc effect. Whereas smaller PD activity related to easy distractor suppression (distractor with an incongruent grip) will lead to a smaller N2pc effect. Increased PD activity has been observed with increased distractor load and it decreases with decreased distractor load (Feldmann-Wüstefeld and Vogel, 2019). We sought to extend the results of affordance based attentional modulation to determine whether seeing objects being gripped in a congruent manner influenced whether objects were selected. While prior studies have presented single items at fixation or peripherally, gripped in either a congruent or incongruent manner, we presented two stimuli (one in the left and the other in the right visual field). Participants were cued with a verb (e.g., drink) and they had to verify whether an object congruent with the cue was present (e.g., cup). Both the target and the distractor object could be grasped correctly or incorrectly to create a 2 × 2 design a 2 (Target Grip Congruency: Congruent grip and incongruent grip) × 2 (Distractor Grip Congruency: Congruent grip and incongruent grip).

## Materials and Methods

### Participants

Fifteen right-handed students from the School of Psychology of the University of Birmingham, who were all unaware of the purpose of the experiment, participated for cash in this study. They were aged between 19 and 26 years and their vision was normal or corrected to normal. Participants provided written consent before participation. The study was approved by the University Ethics Committee.

### Apparatus

A Pentium IV computer with an ATI RAGE PRO 128-MB graphics card controlled the stimulus displays and responses. The task was programmed and run on this computer using E-Prime (Version 1.0; PST, 2002). The stimuli were displayed on a SAMSUNG (Seoul, South Korea) SynchMaster 753s color monitor. Monitor resolution was 1,024 × 768 pixels. The frame rate was fixed at 85 Hz.

### Task and Procedure

**Figure 1:**
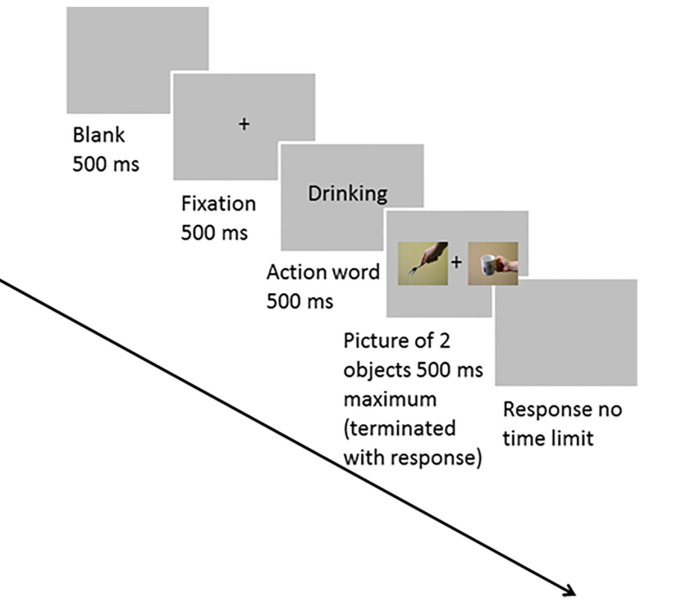
This image shows a trial sequence and example stimuli. Here a target object cup (related to the action word drink) and a distractor object (fork) are congruently gripped (Target congruently gripped and Distractor congruently gripped, TCDC).

**Figure 2:**
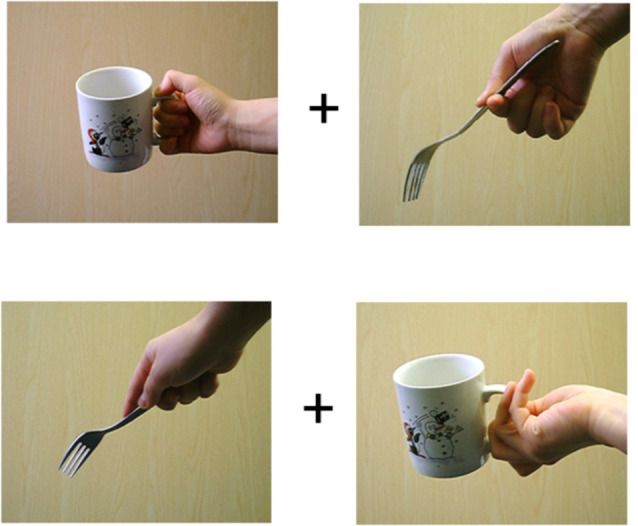
Here example stimuli with congruent (top panel) and incongruent grip (bottom panel) are shown.

The participants sat comfortably at a distance of 70 cm from the computer monitor. At the start of each trial, there was a blank period for 500 ms and a central fixation cross for 500 ms. After this, the action word appeared in the center of the monitor for 500 ms and this was followed by two pictures of objects (each height × width = 6.46° × 7.36°) on either side of the fixation cross. Each was presented at 0.61° from fixation for a maximum of 500 ms followed by a blank screen. The participants could respond when the pictures were on screen or during the blank interval. The trial terminated following a response. Participants performed two blocks of 720 trials each, where the left- or right-hand index finger was used to respond to the presence of the target. In each block, 60 action words were presented. Each action word was presented 12 times followed by a presentation of the object images. Trials were presented randomly in each block. Eight participants responded to the target with their right-hand index finger and vice versa for the remaining participants), pressing an “M” key if a picture in the presentation matched the preceding action word, and the left-hand index finger pressing “Z” key on the keyboard if no picture matched the preceding action word. Figure 1 shows a trial sequence. Objects were held either congruently or incongruently for their use, so in a matching trial the objects presented could be a target object with a congruent grip and a distractor object with a congruent grip (TCDC). The other combinations of presentation could be a target with a congruent grip and a distractor with an incongruent grip (TCDIC); a target with an incongruent grip and a distractor with a congruent grip (TICDC); or a target with an incongruent grip and a distractor with an incongruent grip too (TICDIC). An example of congruent and incongruent grip applied to target and distractor is shown in Figure 2. On the 30% of the trials, neither of the two pictures matched the preceding action word. There was no time limit for making a response. On matching trials, the distractors were never consistent with the cued verb. The same set of target and distractor objects appeared in the left and right visual fields on different trials. A distractor object (e.g., saucepan) paired with a target object (e.g., an ax) on a trial with cue verb chopping was paired with a different object as a distractor (e.g., comb) when it became a target (saucepan) with cue verb chopping. That is the same combinations of target and distractor objects did not appear when different action verbs were used. These objects could be gripped congruently or incongruently.

### EEG Data Processing

Electroencephalogram (EEG) recordings for each participant were taken continuously with Ag/AgCl electrodes from 128 scalp electrode locations. The electrodes were placed according to the 10-5 electrode system (Oostenveld and Praamstra, 2001) using a nylon electrode cap. Vertical eye movements were monitored by a unipolar electrode placed at the *infra* orbital area of the left eye and horizontal eye movements were monitored by bipolar electrodes placed at the outer canthus of the left and right eyes. Common Mode Sense (CMS) and Driven Right Leg (DRL) electrodes were used as references and ground. EEG and electrooculogram (EOG) signals were amplified by BioSemi ActiveTwo amplifiers (Amsterdam, The Netherlands) and sampled at 512 Hz. The continuous EEG recordings were off-line referenced to the average of the left and right mastoids and bandpass filtered between 0.5 and 35 Hz. Continuous EEG signals were segmented into epochs from 200 ms before pictures onset to 800 ms after pictures onset for each of the conditions for each subject only for matched correct trials. The 30% of the trials where none of the pictures matched the action verb were not included in the analysis. Epochs were rejected if the voltage in horizontal eye electrodes exceeded ±50 μV and ±100 μV in any other electrodes. The 200 ms before the onset of the picture stimulus was used as a baseline, and the EEG signals reported were calculated relative to this baseline activity. The N2pc activity was measured in relation to the target position and computed as the average of the contralateral hemispheric activity-an ipsilateral hemispheric activity for the left visual field target and the contralateral hemispheric activity-an ipsilateral hemispheric activity for the right visual field target.

## Results

### Behavioral

**Figure 3:**
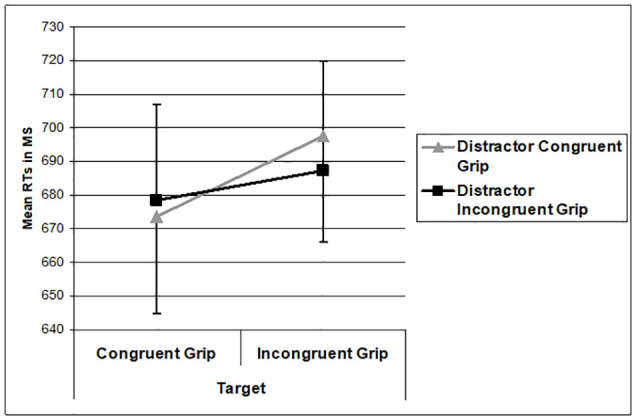
Mean reaction time (RT) performance as a function of the grip applied to the target and distractor. Error bars represent 1 standard error of the mean.

The behavioral data were analyzed for only the trials where the target object matched the action verb and correct responses were made. The 30% of the trials where none of the pictures matched the action verb were not included in the analysis. Reaction times (RTs) were analyzed using a 2 (Target Grip Congruency: Congruent grip and incongruent grip) × 2 (Distractor Grip Congruency: Congruent grip and incongruent grip) repeated measure analysis of variance (RM ANOVA). There was a main effect of target grip congruency where RTs to congruently gripped targets were faster than RTs to incongruently gripped targets (*F*_(1,14)_ = 7.303, *p* = 0.017, $\eta_{P}^{\text{2}}$ = 0.343). RTs were unaffected by distractor congruency (*F*_(1,14)_ = 0.174, *p* = 0.683). The interaction between the target and distractor congruency was not significant (*F*_(1,14)_ = 2.046, *p* = 0.175). The RT data are shown in Figure 3. Overall accuracy across conditions was 88% with no significant main or interaction effect (all *ps* > 0.266).

### ERP Results

#### N2pc Analysis

**Figure 4:**
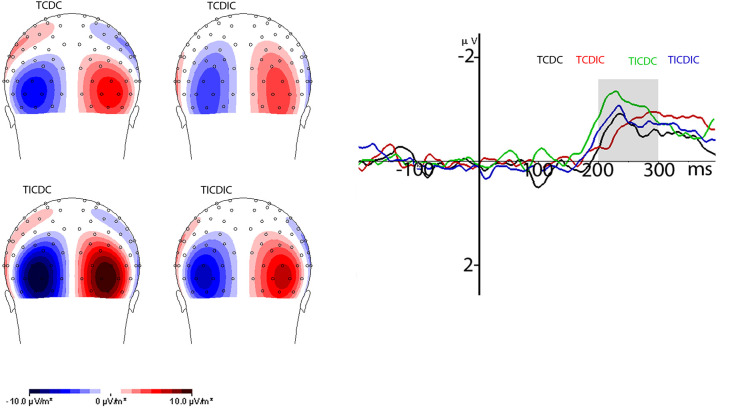
This image shows the grand average contralateral and ipsilateral waveforms locked to the target (left panel) and the grand average N2pc waveform (right panel) for different conditions. The N2pc waveform was computed by subtracting contralateral activity from ipsilateral activity related to the position of the target in the visual field in relation to the electrode position on the scalp. Topography maps are the current source density maps reflecting grand average N2pc activity. Voltage values from the left side (N2pc amplitude) are co-interpolated on the right side of the scalp maps. TCDC, Target congruently gripped and Distractor congruently gripped; TCDIC, Target congruently gripped and Distractor incongruently gripped; TICDC, Target incongruently gripped and Distractor congruently gripped; TICDIC, Target incongruently gripped and Distractor incongruently gripped.

The N2pc component was analyzed at the pooled five posterior and lateral occipital electrodes (POO9h/POO10h, P05h/PO6h, O1/O2 and PO7/PO8) based on the N2pc CSD map where the source of the N2pc activity was observed across the conditions. The N2pc activity occurred at the same source as had been observed earlier in earlier studies (Kiss et al., 2008; Kumar et al., 2009; Telling et al., 2010). The mean amplitude of the N2pc component in the 200–300 ms time window was analyzed. Figure 4 shows posterior contralateral negativity in the 200–300 ms time window on the CSD map (back view, spline interpolation) for the different conditions, grand averaged across participants reflecting the N2pc activity and waveform. N2pc maps were plotted from the difference waveforms for ipsilateral activity subtracted from contralateral activity referenced relative to the side of the target. The resultant map is plotted from co-interpolation of voltage values between the scalp electrodes (Lorenzo-López et al., 2008; Kumar et al., 2009; Telling et al., 2010). Analysis of the N2pc waveform was carried out on the mean amplitudes in a 200–300 ms time window after stimulus onset using a 2 (target congruency) × 2 (distractor congruency) design. This showed a significant main effect of target congruency (*F*_(1,14)_ = 12.074, *p* = 0.004, $\eta_{P}^{\text{2}}$ = 0.463) and a reliable interaction between target congruency and distractor congruency (*F*_(1,14)_ = 4.604, *p* = 0.049, $\eta_{P}^{\text{2}}$ = 0.247). N2pc was overall lower when the target was assigned a congruent grip compared to when the target grip was incongruent. Breakdown of the interaction effect using paired *t-tests* showed that the interaction was due to the N2pc amplitude being lower when the target was congruently gripped and the distractor was incongruently gripped as compared to the other conditions: when the target and the distractor were both congruently gripped (*p* = 0.003), when the target and the distractor were both incongruently gripped (*p* < 0.001) and when the target was incongruently and the distractor congruently gripped (*p* = 0.018). However, the difference between the target congruent distractor congruent condition was not significantly different than the target congruent distractor incongruent (*p* = 0.427) or from the target incongruent distractor incongruent condition (*p* = 0.252). The difference was also not significant between the target congruent distractor incongruent and target incongruent distractor incongruent condition (*p* = 0.665).

In addition to the effects on the amplitude of the N2pc, we also examined how target and distractor congruency-incongruency influenced the onset latency of the N2pc. We used the jackknife method (Miller et al., 1998; Kiesel et al., 2008) with the onset of the N2pc calculated as 50% of the peak N2pc. The results showed a reliable main effect of congruency for both the target (*F*_(1,14)_ = 6.43, *p* = 0.024, $\eta_{P}^{\text{2}}$ = 0.315) and distractor (*F*_(1,14)_ = 5.4, *p* = 0.036, $\eta_{P}^{\text{2}}$ =0.278). However, the influence of congruency on the target and distractor was opposite: shorter N2pc latencies for the congruently gripped distractor (*M* = 203 ms) compared to the incongruently gripped distractor (*M* = 216 ms) and incongruently gripped target (*M* = 199 ms) compared to the congruently gripped target (*M* = 220 ms) were observed. The two-way interaction was not significant (*F*_(1,14)_ = 1.24, *p* = 0.284).

## Discussion

The present study investigated the effect of a visual affordance on attentional selection; in particular, whether a depicted hand grip was applied in a manner that was congruent or incongruent with the action associated with that object. Prior evidence has indicated that the effects of grip congruency are difficult to ignore (Yoon and Humphreys, 2005) and that these effects are associated with early activation of a differential motor response to congruent stimuli which feed-back to influence perceptual coding (Kumar et al., 2012). The present study shows that grip congruency does not only influence the perception of objects but also modulates visual selection. When cued to attend to an object matching a verb label, RTs are speeded when the matching (target) object is gripped correctly. Grip congruency also interacted with the congruency of the grip to distractors when ERPs were measured. Onset latency for the congruently gripped target was delayed compared to the incongruently griped target, whereas the onset latency was earlier for the congruently gripped distractor compared to the incongruently gripped distractor indicating the independent effect of grip congruency on the speed of attentional selection for targets and distractors. Faster attentional deployment to a congruently gripped distractor might be needed to facilitate target processing through inhibition and rapid rejection (Geng and DiQuattro, 2010). Similarly, Hickey et al. (2006) have observed that distractor elicited a lateralized potential earlier than the target-related lateralized potentials suggesting that attention is directed to a salient distractor first before a target is processed. A congruent handgrip applied to a distractor object seems to be a salient feature that needs to be processed and suppressed first. However, we are not sure why there was a faster attentional deployment to an incongruently gripped target.

N2pc amplitude analysis showed that there were clear effects on the N2pc component-a marker of the ease of visual selection (Luck and Hillyard, 1994b; Luck et al., 1997; Telling et al., 2010). Notably, the amplitude of the N2pc was reduced when the target was assigned a congruent grip and the distractor an incongruent grip (TCDIC) compared with the other conditions. The affordance offered by the congruent target and the lack of affordance to the incongruent distractor eased target selection and facilitated maintenance of attention on the target (as observed in the sustained negativity of the N2pc waveform for the target congruent distractor incongruent condition). This observation is consistent with the idea that the N2pc is the sum of the neural resources associated with the target (NT) and distractor processing (PD). Telling et al. (2010) have previously shown that target processing is eased when the competition from distractors is reduced. Indeed, in our study, there is enhanced completion to target selection, which is associated with using a congruent grip to act as a congruently gripped distractor rather than an incongruently gripped distractor. Suppressing a congruently gripped distractor in the present study would have led to a greater PD potential (Feldmann-Wüstefeld and Vogel, 2019) which is likely to have increased the N2pc amplitude (Miller et al., 1998; Kiesel et al., 2008) when a target was selected.

The results from the N2pc analysis also support the basic principles of the pre-motor theory of attention (Rizzolatti et al., 1987, 1994). This theory of attention assumes that there is a shift of attention whenever shared control structures for perception and action are activated (Jeannerod et al., 1995; Prinz, 1997; Grèzes et al., 2003). In an earlier study, Kumar et al. (2013) showed that modulation of mu rhythm de-synchronization was related to grip congruency, with the maximum power of the de-synchronized response emerging 100–150 ms after stimulus onset. This early motor response may facilitate attention to the congruently gripped target, especially when the distractor does not also offer a competing affordance. Handy et al. (2003) also reported that graspable objects automatically capture visual spatial attention (see also Goslin et al., 2012; Freeman et al., 2016). Furthermore, Handy et al. (2003) also showed that tools grab attention only when action-related brain areas are activated, suggesting a close link between the motor-related and attention-related brain areas. However, in a recent behavioral study (Yamani et al., 2016) did not find evidence for prioritization of attentional selection based on affordance properties of the objects. They argued that the object graspability facilitates post-search response processing with little effect on attentional prioritization. Their results are consistent with findings from Lien et al. (2013) who also did not find ERP evidence for an affordance-based effect on attention. Based on our findings albeit using a different paradigm, we demonstrate that attentional capture is modulated by the graspability of the objects.

Kumar et al. (2012) have previously reported effects of grip congruence on the perception related P1 and N1 ERP components when a single object appeared at fixation. In the present study, we extend the findings of grip congruency on target selection when two objects are presented simultaneously gripped congruently or incongruently for their use. Handy et al. (2003) also presented two objects simultaneously in two of the quadrants and a target was superimposed on the objects. Their ERP findings showed an enhanced P1 ERP component associated with increased graspability of the object and the target user. In the current study, we also found that the attention directing N2pc component was modulated by the grip congruency applied to objects. The Graspability of the object has extensively been studied in guiding attention to objects. For example, Garrido-Vásquez and Schubö (2014) showed that attentional allocation was enhanced for graspable objects compared to non-graspable objects. In addition to the graspable nature of objects, attention was further enhanced by the ease of grasp reachability towards those objects (visuospatial attention preferentially allocated to near-space objects). These findings may suggest that ease of orienting visuospatial attention increases as motor experience with objects increases through efficient processing of sensory information at the lateral occipital visual area (Handy et al., 2006). In this context, temporal dynamics of the motor-related brain regions is interesting. Using source analysis of their EEG data, Petit et al. (2006) found that the left motor cortex was significantly activated for natural grip in the time window of 180–280 ms following stimulus presentation. The time window of the N2pc, an ERP component linked to the orientation of spatial attention overlaps with this time window. Based on this understanding, we suggest that the congruent grip applied to objects in our study may have led to increased activity in the motor-related brain areas which further led to efficient attentional allocation. The efficient attentional allocation could have been achieved through sensory gain to congruently grasped objects (for a similar interpretation of the N2pc effect see Luck and Hillyard, 1994a; Zhao et al., 2011). This proposal is consistent with an earlier observation by Craighero et al. (1999) who reported motor facilitation of sensory processing to visually presented objects when participants grasp the object.

In sum, the present study provides evidence that the handgrip applied to objects modulates the ease of visual selection when competing stimuli are present. The handgrip effect has previously been shown to reflect activation of a motor response that is congruent for action (Kumar et al., 2012). Our data provide further support that activation of motor systems can facilitate the visual selection.

## Data Availability Statement

The raw data supporting the conclusions of this article will be made available by the authors, without undue reservation.

## Ethics Statement

The studies involving human participants were reviewed and approved by University Research Ethic Committee, University of Birmingham. The patients/participants provided their written informed consent to participate in this study.

## Author Contributions

SK and GH planned the study. SK collected and analyzed the data. GH and MR provided feedback on the analysis. SK and GH worked on the initial draft of the manuscript. JR provided feedback on the manuscript. GH and JR obtained funding for the project. GH is no more. Obviously, he couldn’t have a say on the final approval. His wife JR has approved the final version. All authors contributed to the article and approved the submitted version.

## Conflict of Interest

The authors declare that the research was conducted in the absence of any commercial or financial relationships that could be construed as a potential conflict of interest.
